# Differentiated instruction in EFL classrooms: teacher beliefs, perceived professional agency, and teacher educator perspectives in Türkiye

**DOI:** 10.3389/fpsyg.2026.1923021

**Published:** 2026-10-01

**Authors:** Hüsnü Alp Balaban, Derya Girgin

**Affiliations:** 1 School of Graduate Studies, Çanakkale Onsekiz Mart University, Çanakkale, Türkiye; 2 Department of Educational Sciences, Çanakkale Onsekiz Mart University, Çanakkale, Türkiye

**Keywords:** differentiated instruction, EFL teaching, inclusive education, learner motivation, perceived professional agency, teacher beliefs, teacher education

## Abstract

**Introduction:**

Differentiated instruction (DI) requires teachers to interpret learner diversity and make adaptive pedagogical decisions within the opportunities and constraints of their professional contexts. This study examined how secondary school English as a Foreign Language (EFL) teachers and teacher educators in Türkiye understood and experienced DI, with particular attention to teacher beliefs, perceived professional agency, and motivational considerations.

**Methods:**

This qualitative interpretive phenomenological study involved semi-structured interviews with 14 EFL teachers and six teacher educators. Data were analyzed through an inductive, interpretive form of thematic analysis informed by Braun and Clarke’s six-phase procedure.

**Results:**

Both participant groups understood DI as an inclusive and ethically responsive approach addressing differences in learner readiness, interests, language proficiency, learning pace, and support needs. Teachers associated DI with stronger student engagement, motivation, participation, and language development, but reported a persistent mismatch between their pedagogical intentions and their capacity to act on them. Large classes, limited planning time, curriculum rigidity, resource inequalities, and examination pressures constrained adaptive decision-making and weakened perceived professional agency. Teacher educators similarly identified a gap between pre-service teachers’ conceptual knowledge and their ability to respond flexibly to learner diversity in authentic classrooms.

**Discussion:**

The findings conceptualize DI as a contextually negotiated practice shaped by the interaction of teacher beliefs, professional agency, motivational commitment, teacher preparation, and institutional conditions. The study highlights the need for practice-based teacher education, sustained professional learning, and organizational environments that enable psychologically responsive and inclusive EFL instruction.

## Introduction

Differentiated instruction (DI), as conceptualized by Tomlinson (2001), involves tailoring content, process, products, and learning environments to accommodate individual learner needs. Globally recognized as a cornerstone of inclusive education, DI promotes equity and engagement in diverse classrooms (Sousa and Tomlinson, 2018; Tomlinson, 2017). Research conducted in diverse national contexts has demonstrated both the promise and the complexity of DI implementation. Studies from the United States, for instance, have documented how teachers struggle to move from theoretical endorsement of DI to consistent classroom application, particularly under conditions of large class sizes and standardized accountability systems (Bondie et al., 2019; Tomlinson, 2015). Research in European contexts, including Belgium and the Netherlands, has similarly found that teachers hold positive attitudes toward differentiation yet lack adequate preparation and institutional support to implement it systematically (Coubergs et al., 2017; Suprayogi et al., 2017). In East Asian settings such as Hong Kong, Wan (2017) found that prospective teachers demonstrate limited self-efficacy for DI despite receiving formal instruction in the approach. These international findings collectively underscore that the challenges of DI implementation are not idiosyncratic but structural, shaped by institutional conditions, curricular demands, and the quality of teacher preparation. Recent evidence further indicates that teachers’ differentiated practices are shaped by their beliefs about student diversity, the organizational environments in which they work, and the extent to which inclusive teaching structures support flexible grouping and instructional adaptation (Bi et al., 2023; Lindner et al., 2021). However, its application in English as a Foreign Language (EFL) classrooms, characterized by unique linguistic and cultural challenges, remains underexplored, particularly in Türkiye, where the intersection of large heterogeneous classes, centralized curricula, and high-stakes testing creates a particularly demanding environment for differentiated practice.

Türkiye’s secondary school EFL classrooms face distinctive challenges including large class sizes, heterogeneous student populations, and standardized testing pressures. English language teaching predominantly employs traditional methods shaped by a one-size-fits-all approach (Kırkgöz, 2007), which fails to meet individual student needs and hinders language learning, especially for students with low motivation (Mardhatillah and Suharyadi, 2023). While the Turkish education system encourages student-centered approaches, DI implementation encounters barriers such as insufficient training, limited resources, and misconceptions about practicality (Gülşen, 2018; Şaban and Atay, 2023). Despite growing recognition of DI’s value, the Turkish EFL literature remains particularly sparse in terms of empirical work drawing on the voices of practicing teachers and teacher educators simultaneously. Gülşen (2018) conducted a meta-synthesis of DI studies in Turkey but found the evidence base thin and methodologically limited. Şaban and Atay (2023) examined DI perceptions in higher education EFL settings, yet secondary school contexts and the role of teacher preparation programs remain largely unaddressed. Recent international EFL research similarly shows that teachers may hold generally positive views of differentiation while remaining underprepared to translate those understandings into systematic classroom practice, particularly when they lack sustained professional learning and context-sensitive curricular support (Grecu, 2023; Shafiei Rezvani Nejad, 2024). This dual gap, namely the absence of practitioner perspectives from both in-service and pre-service settings, constitutes a meaningful empirical lacuna that the present study seeks to fill. To the best of our knowledge, previous research in the Turkish EFL context has not simultaneously examined practicing teachers’ implementation experiences and teacher educators’ perspectives on preparation. Consequently, limited evidence is available regarding how DI-related knowledge is transferred, transformed, or constrained across pre-service preparation and classroom practice. The present study fills this gap by providing cross-level evidence that reveals not only whether DI is understood and implemented, but where and why the connection between teacher preparation and classroom implementation may break down. More importantly, this study moves beyond documenting teachers’ perceptions by examining the continuity between teacher preparation and classroom implementation. Rather than treating pre-service education and in-service practice as separate domains, the study conceptualizes them as interconnected stages within a single professional learning continuum. This perspective allows a more comprehensive understanding of how differentiated instruction is transmitted, transformed, or constrained across educational contexts, thereby providing a conceptual contribution that has received limited attention in previous EFL research.

Adopting differentiated teaching practices is critical for managing classroom diversity and ensuring active participation of all students (Voltz et al., 2010; Yavuz, 2020). However, research on teacher competencies and perceptions regarding DI in Türkiye remains limited, with insufficient information about teacher educators’ processes for preparing prospective teachers. DI aligns with constructivist theories emphasizing tailored teaching to address varied student readiness, interests, and learning profiles. In EFL classrooms, where learners have diverse linguistic proficiencies, DI offers a means of responding to learner diversity while fostering active engagement (Chien, 2012; Tomlinson, 1999).

Theoretical foundations for DI draw from multiple perspectives. Vygotsky’s zone of proximal development (ZPD) concept emphasizes providing instruction slightly beyond students’ current independent performance levels while offering appropriate support (Vygotsky, 1978). This scaffolded approach inherently requires differentiation, as students’ ZPDs vary based on prior knowledge, language proficiency, and cognitive development. Effective DI identifies each student’s ZPD and provides targeted instruction and support within that zone, maximizing learning potential.

Cognitive load theory suggests that working memory limitations constrain learning, particularly when tasks exceed cognitive capacity (Sweller et al., 2011). In EFL contexts, language processing demands substantial cognitive resources, especially for beginners. Differentiation can optimize cognitive load by adjusting task complexity, providing support structures, and sequencing instruction to build gradually from simple to complex. For advanced learners, reducing unnecessary supports challenges them appropriately without overwhelming less proficient students. This cognitive load perspective helps teachers make principled decisions about when and how to differentiate content and process.

Although differentiated instruction has traditionally been conceptualized as an instructional framework focused on adapting teaching to learner differences (Tomlinson, 2001), the present study approaches DI as a context-dependent pedagogical practice shaped by the interaction of teacher beliefs, institutional conditions, and professional preparation. From this perspective, teachers’ implementation decisions emerge through the continuous negotiation of pedagogical knowledge, curriculum expectations, available resources, and learner diversity. Accordingly, understanding differentiated instruction requires examining not only classroom strategies but also the broader educational contexts that enable or constrain teachers’ capacity to differentiate instruction. This conceptual perspective provides the theoretical lens through which the findings of the present study are interpreted. Contemporary inclusive pedagogy further suggests that differentiation should be evaluated not only in relation to academic access but also through students’ social inclusion, emotional well-being, and academic self-concept. Evidence indicates that students who perceive stronger inclusive teaching practices also report more positive academic, social, and emotional inclusion (Alnahdi et al., 2022).

## Study aims

This research examines English language teachers’ and teacher educators’ perceptions of differentiated instructional design in Balıkesir and Çanakkale provinces, aiming to develop recommendations for integrating DI into English lessons. From an educational psychology perspective, the study examines how teachers’ beliefs, perceived professional agency, and experiences of institutional constraint shape their willingness and capacity to enact differentiated instruction. Specific objectives include: (a) determining secondary school English teachers’ opinions on differentiated instructional design, (b) determining teacher educators’ perspectives in English Language Education departments, (c) identifying similarities and differences between these groups’ opinions, and (d) developing suggestions for DI integration in English lessons.

## Study significance

This phenomenological study reveals differentiated teaching perspectives in EFL contexts based on practitioners’ opinions and experiences. The study provides educators and curriculum development experts with information about the effectiveness, strengths, and weaknesses of DI implementation. It also raises awareness among teachers and teacher educators of differentiated teaching in foreign language instruction and provides information on implementing the methods and techniques of this approach, potentially motivating educators to revitalize DI practices. Beyond its practical implications, the study contributes theoretically by illustrating differentiated instruction as a dynamic process shaped through the interaction of teacher beliefs, institutional structures, and teacher education experiences. The findings therefore extend existing discussions of differentiated instruction from a predominantly classroom-focused perspective toward a broader teacher education perspective, highlighting how preparation and implementation mutually influence one another.

## Literature review

### Differentiated instruction

Differentiated instruction redesigns and adapts teaching processes considering students’ individual learning needs, readiness levels, interests, and learning styles (Tomlinson, 2001). This approach overcomes traditional teaching’s one-size-fits-all mentality, delivering flexible, student-centered education. The ultimate aim is tailoring instruction to ensure each learner receives opportunities to succeed according to their unique profile.

According to Heacox (2012), DI operates through four primary elements: content, process, product, and learning environment. Content differentiation involves diversifying instructional materials and information conveyed to students, using various reading resources offering multiple perspectives to appeal to different interests and background knowledge. Process differentiation adjusts how students interact with content, including flexible grouping, tiered assignments, and learning centers (Tomlinson and Imbeau, 2010). Product differentiation allows students to demonstrate learning through various formats, including presentations, written reports, creative projects, and performances, ensuring assessment methods align with individual strengths (Gregory and Chapman, 2013). Learning environment differentiation considers physical, psychological, and social classroom aspects, creating safe, flexible spaces accommodating diverse learning preferences (Sousa and Tomlinson, 2018).

### Teacher training and professional development

Research demonstrates that DI effectiveness depends heavily on teacher preparedness. Studies reveal many educators feel inadequately prepared to implement differentiation, citing insufficient pre-service and in-service training (Roy et al., 2013; Santangelo and Tomlinson, 2012). Effective teacher education programs must integrate DI principles throughout curricula rather than treating them as isolated topics (Wan, 2017). Recent studies support this integrated approach, showing that prospective teachers’ differentiated instruction competencies are strengthened when teacher educators model differentiation, connect it with inclusive frameworks such as Universal Design for Learning, and provide repeated opportunities for applied design, enactment, and reflection (Griful-Freixenet et al., 2021; Nketsia et al., 2024). This requires modeling differentiated practices in teacher education courses, providing opportunities for prospective teachers to design and implement differentiated lessons, and fostering reflective practice about meeting diverse learner needs (Coubergs et al., 2017).

Professional development opportunities for in-service teachers must be sustained, practical, and school-based to effect meaningful change (Suprayogi et al., 2017). One-time workshops prove insufficient; teachers need ongoing support, coaching, and opportunities to collaborate with peers in applying DI strategies (Dixon et al., 2014). A recent synthesis similarly concluded that effective DI preparation and professional development tend to be longitudinal and comprehensive and incorporate active learning, collaboration, and structured reflection rather than relying on short-term knowledge transmission (Langelaan et al., 2024). Vygotsky’s sociocultural theory underscores the importance of collaborative learning and scaffolding, highlighting the need for professional communities of practice (Lave and Wenger, 1991).

Teachers’ capacity to differentiate is also influenced by motivational and psychological characteristics. In particular, self-efficacy and enthusiasm appear to support teachers’ willingness to invest the additional planning, monitoring, and adaptive decision-making required for differentiated instruction (Kalinowski et al., 2024). This perspective suggests that professional development should address not only teachers’ procedural knowledge but also their confidence and motivation to enact DI under demanding classroom conditions.

### Barriers to implementation

Despite DI’s potential benefits, implementation faces considerable challenges. Large class sizes make individualizing instruction difficult (Goddard et al., 2015). Standardized curricula and high-stakes testing pressures constrain teacher flexibility (Tomlinson, 2015). Time limitations, both for planning and instruction, pose significant barriers (Suprayogi et al., 2017). Limited resources, including materials and technology, restrict differentiation possibilities (Bondie et al., 2019). Recent research indicates that curricular mandates, limited preparation time, insufficient professional development, and restricted opportunities for teacher collaboration may jointly prevent teachers from adapting otherwise useful curricular resources to heterogeneous classrooms (Grecu, 2023).

Beyond practical constraints, attitudinal and conceptual barriers exist. Some teachers hold misconceptions about DI, viewing it as creating individualized lessons for every student rather than flexible grouping and varying approaches (Tomlinson, 2015). Others question whether DI compromises academic rigor or unfairly advantages some students (Watts-Taffe et al., 2012). These beliefs underscore the importance of addressing both practical and philosophical aspects of DI in teacher education and professional development.

The EFL context presents distinctive considerations for differentiated instruction. Language classrooms inherently involve learners at varying proficiency levels, from beginners struggling with basic communication to advanced learners capable of nuanced expression (Tomlinson, 1999). This proficiency heterogeneity requires differentiation not only by general learning characteristics but specifically by linguistic accessibility. Teachers must scaffold language input, adjust task complexity, and provide varied support structures based on students’ current interlanguage development.

Motivation represents another critical dimension in EFL differentiation. Unlike content areas where motivation may stem from intrinsic interest in subject matter, language learning motivation involves complex factors including integrative motivation (desire for cultural connection), instrumental motivation (pragmatic goals), and self-efficacy beliefs (confidence in language learning ability). Differentiated approaches can support engagement by providing varied materials, tasks, and learning opportunities that respond to learners’ readiness, interests, and proficiency levels (Chien, 2012). Providing culturally relevant content, authentic communication opportunities, and achievable yet challenging tasks can support diverse motivational profiles.

Cultural and sociolinguistic considerations further complicate EFL differentiation. Students bring varied cultural backgrounds influencing their communication styles, learning preferences, and classroom participation patterns. In the Turkish context, students’ prior English exposure varies dramatically based on family socioeconomic status, geographic location, and educational opportunities. Some students access English through travel, private tutoring, or immersive experiences, while others rely solely on school instruction. This experiential diversity necessitates differentiation addressing not only language skills but also cultural knowledge and metalinguistic awareness.

Taken together, previous studies have demonstrated the pedagogical benefits of differentiated instruction and identified numerous implementation barriers across educational settings. Nevertheless, considerably less attention has been devoted to understanding how teacher education experiences influence later classroom implementation, particularly within EFL contexts characterized by centralized curricula and examination-oriented educational systems. Addressing this gap requires examining both teacher educators and practicing teachers simultaneously so that continuities and discontinuities between preparation and practice can be better understood.

This study addresses the following research questions:

1. How do secondary school EFL teachers conceptualize and implement differentiated instruction in their classrooms?
2. What are teacher educators’ perspectives on preparing pre-service teachers for DI implementation?
3. What challenges do both groups identify in DI implementation, and what solutions do they propose?
4. How do teachers’ and teacher educators’ perspectives converge and diverge regarding DI in EFL contexts?

By addressing these research questions, the present study seeks not only to describe participants’ experiences but also to explain how contextual, institutional, and pedagogical factors interact to shape differentiated instruction in Turkish EFL classrooms. Accordingly, the study aims to generate implications that are relevant for both differentiated instruction scholarship and teacher education research.

## Method

### Research design

This qualitative study employed a phenomenological design to explore lived experiences and perceptions of differentiated instruction among EFL teachers and teacher educators. Phenomenological research examines how individuals make sense of their experiences, seeking to understand the essence of phenomena through participants’ perspectives (Creswell and Poth, 2018). This approach was appropriate for investigating complex, multifaceted perceptions of DI implementation in authentic educational contexts. More specifically, the study adopted an interpretive phenomenological orientation informed by van Manen’s (2016) hermeneutic phenomenology, which emphasizes understanding the meaning of lived experience through attentive interpretation rather than purely descriptive reduction. This orientation was preferred over purely descriptive traditions (e.g., Giorgi, 2009) because it accommodates the inherently interpretive character of understanding teacher beliefs and practice, recognizing that participants’ accounts of DI are always already shaped by professional context, institutional pressures, and prior pedagogical understanding.

Consistent with the interpretive phenomenological orientation of the study, the research process incorporated several principles that guided data collection and analysis. First, the researchers engaged in reflexive bracketing throughout the study. Prior to data collection, both researchers documented their professional assumptions, previous teaching experiences related to differentiated instruction, and expectations regarding what teachers and teacher educators might report. Rather than claiming the complete suspension of prior assumptions, the researchers engaged in reflexive bracketing by identifying, documenting, and critically examining how their prior experiences might shape interpretation. The reflective memos served as an ongoing reference point throughout the analysis, helping the researchers remain aware of their pre-understandings and critically examine their influence on the interpretive process. Regular analytical discussions and peer-debriefing meetings were also used to examine interpretations that might have reflected researcher assumptions rather than participants’ meanings. These procedures supported interpretive openness while recognizing that the researchers’ prior knowledge and disciplinary backgrounds could not be entirely separated from the process of understanding participants’ lived experiences.

The analysis was guided by the phenomenological concept of intentionality, the principle that consciousness is always directed toward something, an object or phenomenon. In this study, participants’ accounts were understood not as neutral reports of external events but as constituted experiences: their perceptions of DI were shaped by their professional contexts, values, and prior encounters with the approach. Accordingly, the researchers attended not only to what participants said about DI but to how they oriented toward it, whether as a practical tool, an ethical obligation, an institutional expectation, or a source of professional tension. These intentional structures were treated as analytically significant rather than incidental.

The analysis aimed to identify essential themes, defined as structures of meaning that persisted across individual accounts rather than remaining idiosyncratic to particular participants. Through iterative reading, coding, and discussion, the research team sought convergences in how participants experienced the phenomenon of differentiated instruction: what it meant to them, what it demanded, what it promised, and where it fell short. These convergent structures constituted the essence of the phenomenon as experienced by this group of EFL practitioners, forming the basis of the thematic framework presented in the results.

### Participants

Participants were recruited through purposeful sampling from Balıkesir and Çanakkale provinces. These provinces were selected because both include universities with established English Language Teaching departments as well as diverse secondary school types, providing access to participants working within varied institutional settings. Geographical proximity of the two provinces also facilitated scheduling and member-checking procedures. The sample comprised 14 secondary school English teachers, including 11 female and 3 male, working in state and private secondary schools in Balıkesir and Çanakkale. Their professional experience was distributed across four categories: 6–10 years (*n* = 1), 16–20 years (*n* = 4), 21–25 years (*n* = 5), and 26–30 years (*n* = 4). The study also included six teacher educators, including three women and three men, working in English Language Teaching departments in Balıkesir and Çanakkale. Their higher education experience ranged across the categories of 11–15 years (*n* = 1), 16–20 years (*n* = 1), 21–25 years (*n* = 3), and 31–35 years (*n* = 1). Detailed participant characteristics are presented in Tables 1, 2. Inclusion criteria required teachers to have a minimum of 5 years of EFL teaching experience in secondary schools with learner diversity, and teacher educators to be actively involved in methodology or practicum courses in EFL preparation programs. Data collection continued until sufficient experiential and thematic depth was achieved. No substantively new meaning structures emerged in the final three teacher interviews or the final teacher educator interview. The adequacy of the sample was also supported by the principle of information power (Malterud et al., 2016), as participants possessed substantial professional experience directly relevant to the research phenomenon and generated rich, information-dense interview data. All participants were adult professionals and provided their own voluntary informed consent prior to participation. Ethical approval and institutional research permission were obtained before data collection commenced.

**Table 1:** Demographic characteristics of teacher participants.

Participant	Gender	Educational background	Working period	Service city	Institution of service
T1	Female	Bachelor’s	16–20 years	Çanakkale	Private
T2	Male	Bachelor’s	16–20 years	Çanakkale	State
T3	Female	Bachelor’s	21–25 years	Balıkesir	State
T4	Male	Bachelor’s	26–30 years	Balıkesir	State
T5	Female	Bachelor’s	26–30 years	Balıkesir	State
T6	Female	Bachelor’s	26–30 years	Balıkesir	State
T7	Male	Master	16–20 years	Çanakkale	State
T8	Female	Bachelor’s	21–25 years	Çanakkale	State
T9	Female	Bachelor’s	21–25 years	Çanakkale	State
T10	Female	Bachelor’s	21–25 years	Çanakkale	State
T11	Female	Bachelor’s	6–10 years	Çanakkale	State
T12	Female	PhD	21–25 years	Çanakkale	State
T13	Female	Bachelor’s	26–30 years	Çanakkale	State
T14	Female	Master	16–20 years	Çanakkale	State
14 Participants	11 Females 3 Males	11 Bachelor’s 2 Master 1 PhD	1 p. 6–10 years 4 p. 16–20 years 5 p. 21–25 years 4 p. 26–30 years	4 p. Balıkesir 10 p. Çanakkale	1 Private 13 State

**Table 2:** Demographic characteristics of teacher educator participants.

Participant	Gender	academic title	Working period	Service city	Experience in a foundation related to ministry of national education (private and/or state)
A1	Female	Instructor	16–20 years	Çanakkale	No
A2	Male	Assistant professor	21–25 years	Çanakkale	Yes
A3	Male	Associate professor	21–25 years	Çanakkale	No
A4	Female	Professor	31–35 years	Çanakkale	No
A5	Female	Associate professor	21–25 years	Balıkesir	Yes
A6	Male	Assistant professor	11–15 years	Balıkesir	Yes
6 Participants	3 Females 3 Males	1 Instructor 2 Assistant Professor 2 Associate Professors 1 Professor	1 p. 11–15 years 1 p. 16–20 years 3 p. 21–25 years 1 p. 31–35 years	4 p. Çanakkale 2 p. Balıkesir	3 Yes 3 No

### Data collection

Data were collected through semi-structured interviews between September and October 2025, at the beginning of the 2025-2026 academic year. Ethical approval was obtained on October 3, 2024, followed by the required institutional research permission on January 2, 2025. Accordingly, all interviews were conducted after both ethical approval and institutional permission had been granted. Separate interview protocols were developed for teachers and teacher educators, each containing 12–15 open-ended questions exploring participants’ understanding of DI, implementation strategies, perceived benefits and challenges, and suggestions for improvement. Sample questions for teachers included: How do you define differentiated instruction? What methods do you use to address diverse learner needs? and What challenges do you face in implementing DI? Teacher educator questions focused on how they prepare pre-service teachers for DI, their observations of student teachers’ DI skills, and systemic barriers in teacher education programs. Tables 3, 4 present the main guiding questions for each protocol; the stated total of 12–15 questions reflects the inclusion of additional probing and follow-up questions used during interviews to elicit elaboration and contextual depth.

**Table 3:** Semi-structured interview questions for teachers.

The original questions	English version of the questions
Farklılaştırılmış öğretimi nasıl tanımlarsınız ve EFL bağlamında bunun önemini nasıl değerlendirirsiniz?	How would you define differentiated instruction and how would you evaluate its importance in the context of EFL (teaching English as a foreign language)?
Öğrencilerinizin farklı öğrenme ihtiyaçlarını karşılamak için hangi öğretim yöntem, teknik ve/veya stratejilerini kullanıyorsunuz? Örnekler verebilir misiniz?	What teaching methods, techniques and/or strategies do you use to meet the different learning needs of your students? Can you give examples?
Farklılaştırılmış öğretim tasarımı yaparken dikkate aldığınız anahtar faktörler nelerdir?	What are the key factors you consider when designing differentiated instruction? (For example, students’ language level, learning styles, interests and abilities, readiness, etc.)
Farklılaştırılmış öğretimin öğrencilerin dil gelişimine etkileri hakkında ne düşünüyorsunuz?	What do you think about the effects of differentiated instruction on students’ language development? What positive or negative effects have you observed?
Meslekî gelişiminiz sırasında farklılaştırılmış öğretim ile ilgili eğitim aldınız mı?	Have you received training on differentiated instruction during your professional development? How do you think this training is reflected in your teaching practices?
Farklılaştırılmış öğretimi uygularken karşılaştığınız en büyük zorluklar nelerdir?	What are the biggest challenges you face while implementing differentiated instruction? What methods have you tried/are you trying to overcome these difficulties?
Eklemek istediğiniz başka bir şey var mı?	Is there anything else you would like to add? If so, please specify.

**Table 4:** Semi-structured interview questions for teacher educators.

The original questions	English version of the questions
Farklılaştırılmış öğretimi nasıl tanımlarsınız ve farklılaştırılmış öğretim yaklaşımının öğretmen yetiştirme programlarında yer almasının önemini nasıl değerlendirirsiniz?	How do you define differentiated instruction and how do you evaluate the importance of including the differentiated instruction approach in teacher training programs?
Farklılaştırılmış öğretim konusunda öğretmen adaylarına nasıl bir eğitim veriyorsunuz?	What kind of training do you provide to teacher candidates about differentiated instruction? What teaching methods, techniques and/or strategies do you highlight?
Öğretmen adaylarının farklılaştırılmış öğretim tasarımı yapabilme becerilerini nasıl değerlendiriyorsunuz?	How do you evaluate teacher candidates’ ability to design differentiated instruction? What are the areas where they have the most difficulty in this regard?
Farklılaştırılmış öğretimin EFL bağlamında öğretmen adaylarının mesleki gelişimine katkılarını nasıl görüyorsunuz?	How do you see the contributions of differentiated instruction to the professional development of prospective teachers in the context of EFL (teaching English as a foreign language)?
Öğretmen eğitiminde farklılaştırılmış öğretim yaklaşımının uygulanması sırasında karşılaşılan zorluklar nelerdir?	What are the challenges encountered during the implementation of the differentiated instruction approach in teacher education and how do you propose to overcome these challenges?
Eklemek istediğiniz başka bir şey var. mı?	Is there anything else you would like to add? If so, please specify.

Interviews lasted 45–70 min (*M* = 56 min), were conducted in Turkish, audio-recorded with permission, and transcribed verbatim. Transcripts were reviewed for accuracy and returned to participants for member checking, enhancing trustworthiness. All 20 participants (14 teachers, 6 educators) returned transcript feedback within 3 weeks: 18 confirmed accuracy without changes, while two requested minor phrasing corrections that were incorporated. A second member-checking cycle was subsequently conducted to verify preliminary themes: 12 participants voluntarily reviewed theme summaries, and while no themes required substantive revision, four participants contributed additional contextual detail that enriched interpretive notes. Participant feedback largely confirmed the credibility of the emerging interpretations. Rather than requiring substantive modification of the thematic structure, participants’ comments provided additional contextual clarification that further enriched the researchers’ interpretive understanding of the phenomenon. This iterative member-checking process strengthened the credibility of the findings by ensuring that analytical interpretations remained grounded in participants’ own understandings.

### Data analysis

The data were analyzed through an inductive, interpretive form of thematic analysis informed by Braun and Clarke’s (2006) six-phase procedure. The term inductive here refers to the data-driven orientation of the coding process and should not be conflated with inductive content analysis as a distinct methodological approach (e.g., Hsieh and Shannon, 2005); the present study adheres throughout to Braun and Clarke’s (2006) thematic analysis framework. Although thematic analysis was developed outside phenomenological research traditions, it was considered appropriate for the present study because it enabled the systematic identification and interpretation of shared patterns of meaning across participants’ accounts while preserving sensitivity to their lived experiences (Finlay, 2011). The analysis was inductive in that codes and themes were developed from participants’ accounts rather than imposed through a predetermined coding framework. It was interpretive in that the researchers attended not only to what participants reported about differentiated instruction but also to how they understood and experienced it within their professional and institutional contexts. Rather than producing exhaustive individual phenomenological descriptions, the analysis sought to identify common structures of meaning across the dataset while retaining the contextual richness of participants’ experiences. The analytical process involved six phases: (a) familiarization with the data through repeated reading, (b) systematic generation of initial codes, (c) development of potential themes by grouping related codes, (d) review of themes in relation to the coded extracts and the entire dataset, (e) definition and naming of themes, and (f) production of the final analytical account. Two researchers independently coded 30% of the transcripts, achieving an inter-coder agreement rate of 89% and a Cohen’s kappa coefficient of 0.85, indicating strong agreement. The 89% figure represents raw percentage agreement—that is, the proportion of coding units assigned identical codes by both researchers—whereas the Cohen’s kappa coefficient corrects for chance agreement, providing a more conservative estimate of coding consistency. Coding discrepancies were discussed collaboratively until consensus was reached. When disagreements reflected alternative but equally plausible interpretations, the researchers revisited the original interview transcripts and their analytical memos before making final coding decisions. This collaborative process strengthened the consistency and credibility of the thematic interpretations while maintaining sensitivity to participants’ lived experiences.

The analysis revealed interconnected themes and categories reflecting cognitive, affective, pedagogical, and contextual aspects of DI. MAXQDA software facilitated data organization and coding. To ensure trustworthiness, we employed multiple strategies: prolonged engagement with data, peer debriefing, member checking, and maintaining an audit trail documenting analytical decisions (Lincoln and Guba, 1985). Peer debriefing was conducted with an independent colleague external to the project in three structured sessions across the analysis period; all commentary was documented in analytical memos and used to scrutinize emerging interpretations. During the 30% inter-coder reliability check, eleven initial coding disagreements arose; all were resolved through structured discussion without recourse to a third-party adjudicator. The audit trail comprised codebooks, iterative thematic maps, and reflexive memos recording the rationale for key analytical decisions, enabling retrospective verification of the interpretive process.

Reflexivity played an important role throughout analysis. Researchers maintained analytic memos documenting interpretive decisions, emerging insights, and questions arising during coding. Regular research team meetings involved discussing coded segments, negotiating theme boundaries, and ensuring interpretations remained grounded in data rather than imposing predetermined frameworks. This iterative, dialogic process enhanced analysis rigor and trustworthiness. Negative case analysis searched for data contradicting emerging themes, ensuring interpretations accounted for complexity and variation rather than forcing artificial consensus. Collectively, the procedures described above, including researcher reflexivity, member checking, peer debriefing, intercoder agreement, negative case analysis, and maintenance of an audit trail, addressed Lincoln and Guba’s (1985) criteria of credibility, dependability, confirmability, and transferability.

## Results

### Teachers’ perspectives on differentiated instruction

Analysis of teacher interviews revealed four overarching themes regarding DI conceptualization: (a) understanding of differentiated instruction, (b) key features, (c) importance in EFL contexts, and (d) determinants and components (see Figure 1).

**Figure 1:**
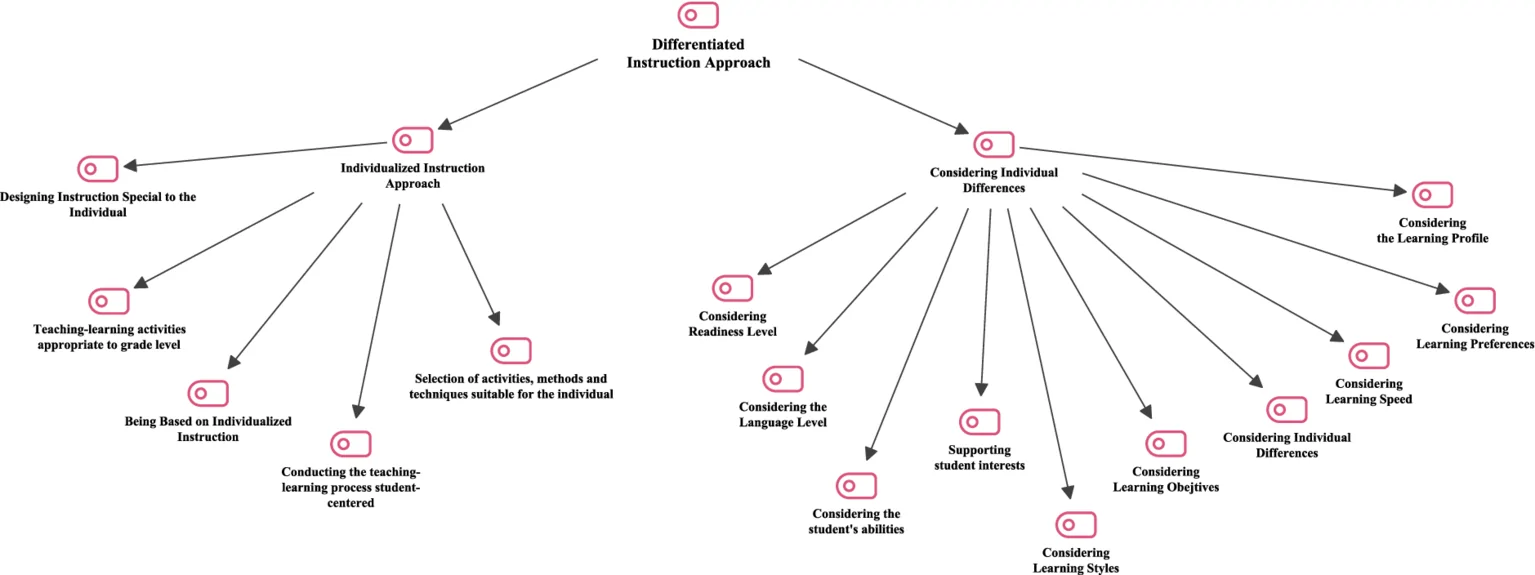
Differentiated instruction approach: Theme 1 and related categories and codes. This thematic map displays teachers’ understanding of DI with two main categories.

#### Understanding of differentiated instruction

Teachers consistently defined DI as a student-centered, inclusive pedagogical approach addressing students’ varied readiness levels, interests, and learning styles. Two primary categories emerged: considering individual differences and individualized instruction approach. Teachers emphasized preparing flexible lesson plans sensitive to diverse needs, interests, and learning paces. They positioned DI as responsive to heterogeneous classes shaped by different learning profiles, interests, and motivation levels. The individualized instruction approach reflected teachers’ commitment to designing strategies, materials, and content according to each student’s unique needs and characteristics, viewing themselves as facilitators continually restructuring instruction according to individual learning profiles while maintaining curriculum consistency.

These perceptions carry clear phenomenological significance: teachers did not simply report using DI techniques but described experiencing it as a fundamentally different orientation toward their students. As one participant expressed, “I no longer see the class as a single group I need to get through a topic. I see thirty different starting points, and my job is to build as many pathways as I can” (T9). Another teacher articulated a similar intentional stance: “Differentiation changed how I look at failure. If a student is not progressing, I stop asking what is wrong with the student and start asking what is wrong with my lesson” (T3). These accounts reflect a constituted understanding of DI not merely as a pedagogical strategy but as an epistemic shift in how teachers perceived their professional responsibility.

#### Key features of differentiated instruction

Teachers identified two key feature categories: design and planning dimension, and innovative and dynamic structure. Regarding design, teachers positioned lesson planning at DI’s center, emphasizing systematic curriculum integration through conscious design processes adaptable to each classroom context and student profile. The innovative and dynamic structure category revealed teachers’ adoption of DI as a flexible, inclusive approach creating enriched learning environments. Teachers described DI as a dynamic model fostering inclusive atmospheres accommodating diverse learner needs (see Figure 2).

**Figure 2:**
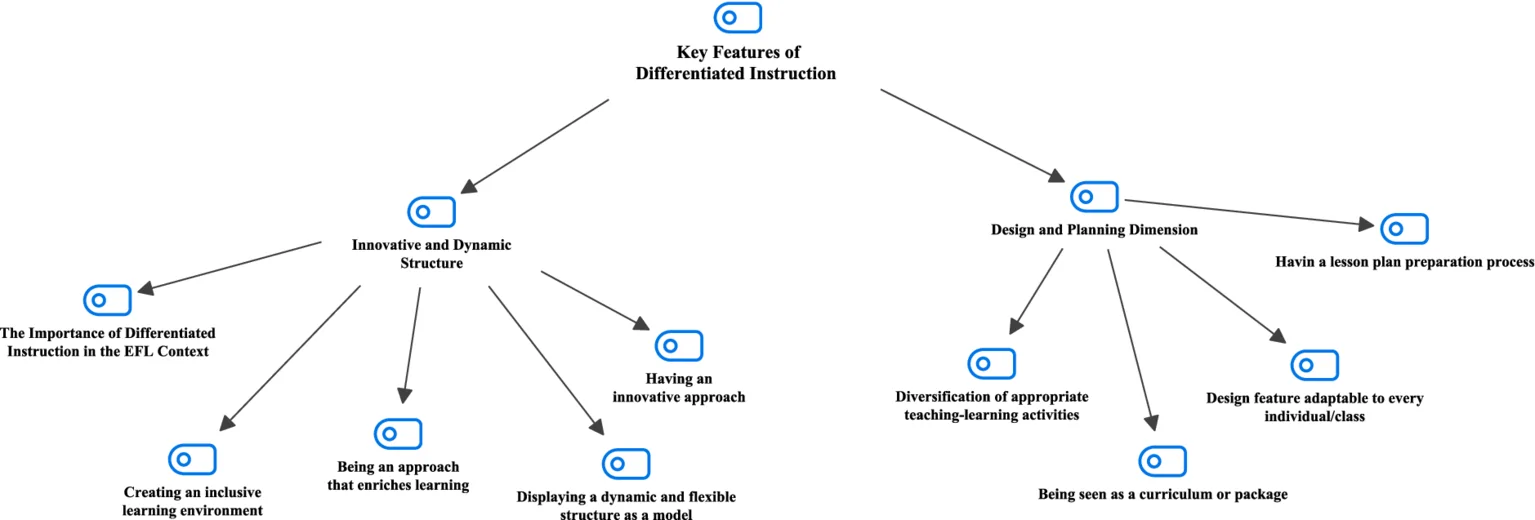
Differentiated instruction approach: Theme 2 and related categories and codes. This figure illustrates key features: design/planning dimension and innovative/dynamic structure.

#### Importance in EFL contexts

Teachers identified three contribution categories: learning process, student gains, and teaching environment. Participants stated DI increases student motivation, supports permanent learning, and enhances effectiveness in foreign language teaching. Teachers believed DI helps students progress at their own pace, supporting holistic development through increased course interest, language development support, participation, and academic success. Some teachers emphasized DI strengthens teacher-student interaction and contributes to flexible, adaptable classroom cultures, creating inclusive and dynamic learning atmospheres. These perceptions of benefit extended beyond pedagogical observations. Participants described experiences such as previously disengaged students beginning to participate and struggling learners gaining visible confidence as meaningful professional moments that strengthened their commitment to differentiated instruction.

Participant accounts provided concrete phenomenological texture to these general perceptions. One teacher described the relational dimension of this transformation: “When I started using differentiated tasks for speaking activities, students who never volunteered suddenly started raising their hands. It was not because the task was easier; it was because, for the first time, they felt it was designed for someone like them” (T11). Another participant captured how DI reshaped the meaning of classroom success: “Some of my strongest students academically told me at the end of the year that they actually enjoyed English for the first time. The challenge was calibrated to them, not to a fictional average” (T7). These accounts point to a constituted experience in which DI altered not just instructional delivery but the affective dimensions of both teaching and learning (see Figure 3).

**Figure 3:**
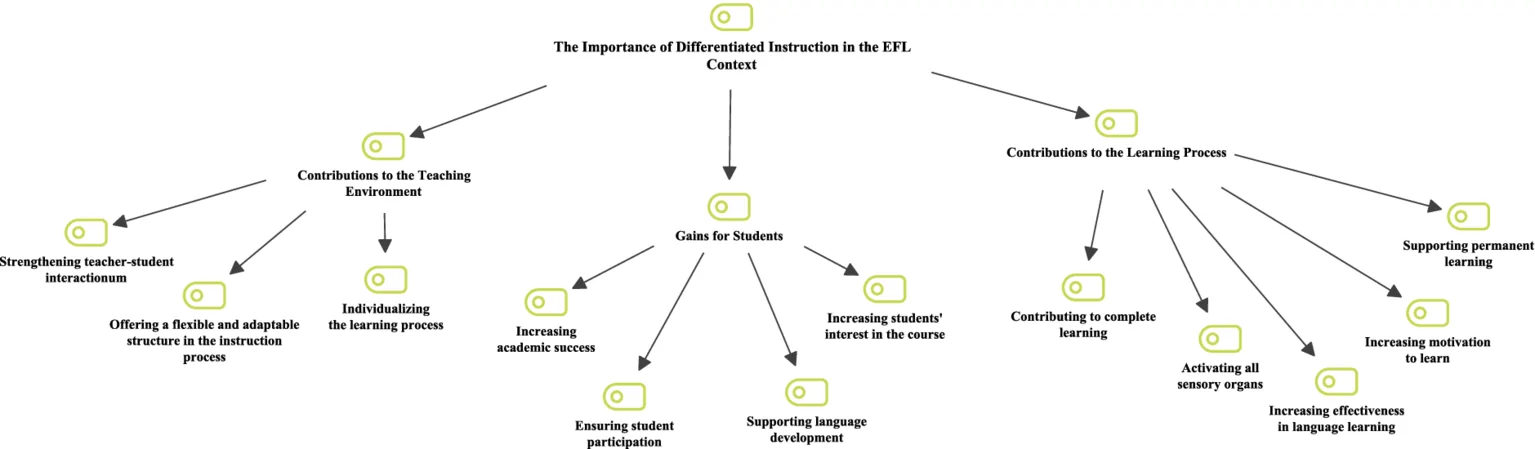
Differentiated instruction approach: Theme 3 and related categories and codes. This map shows three contribution categories: learning process, student gains, teaching environment.

#### Determinants and components

Teachers identified two component groups: student variables (readiness, interests, abilities, learning styles, motivation, language level) and instructional variables (objectives, content diversity, process differentiation, product differentiation). Teachers emphasized understanding and balancing these variables is critical for effective differentiation in foreign language lessons, revealing comprehensive understanding addressing the whom, what, and how of instruction holistically (see Figure 4).

**Figure 4:**
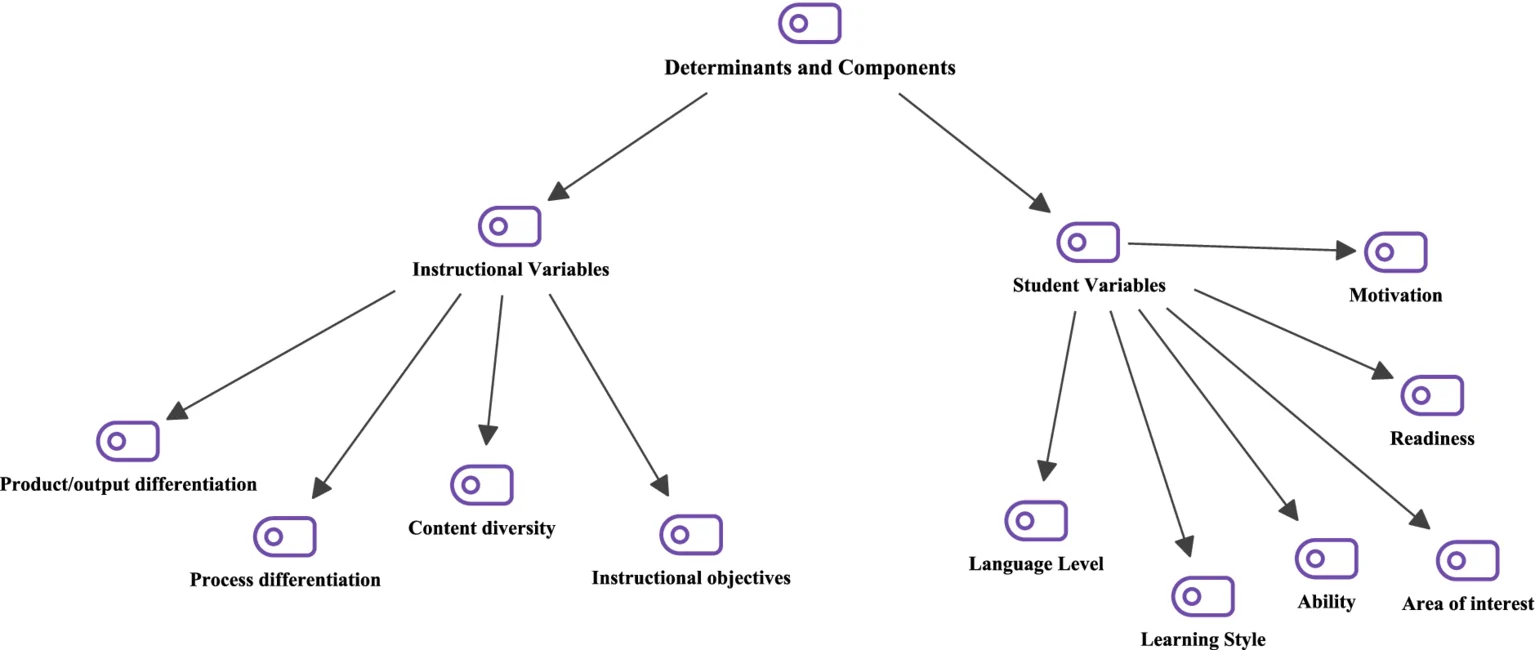
Differentiated instruction approach: Theme 4 and related categories and codes. This figure displays student variables and instructional variables components.

### Implementation methods and strategies

Five themes emerged regarding implementation: (a) instruction adapted to learning styles and individual differences, (b) content and material diversity, (c) evaluation and feedback practices, (d) classroom management and process design, and (e) inclusive and holistic design approach. Teachers emphasized multisensory learning activities combining visual, auditory, and kinesthetic elements. They used flashcards, videos, smartboards, songs, and physical games to accommodate different learning preferences. Teachers adapted instruction according to students’ language proficiency levels, readiness, and developmental characteristics, organizing activities at different levels to reduce disengagement.

Teachers provided specific examples of multisensory implementation. Visual learners benefited from graphic organizers, vocabulary charts, color-coded grammar rules, and video materials. One teacher described using picture stories for narrative writing, where students sequenced images before composing texts. Another teacher explained creating visual timelines for teaching verb tenses, helping students conceptualize temporal relationships spatially. Digital tools like interactive whiteboards enabled dynamic visual presentations accommodating diverse visual learning needs.

Auditory approaches incorporated songs, chants, podcasts, and pronunciation drills. Teachers utilized rhythm and melody to facilitate vocabulary retention and grammar pattern acquisition. One teacher described teaching irregular verb forms through rhythmic chants, noting students remembered patterns more easily when associated with musical elements. Another teacher used authentic listening materials, including weather forecasts, radio advertisements, interviews, exposing students to varied accents and speech rates while building listening comprehension.

Kinesthetic activities involved total physical response (TPR) techniques, role-plays, simulations, and hands-on projects. Teachers described students acting out dialogues, creating physical representations of grammatical concepts, and engaging in communicative games requiring movement. One teacher explained using scavenger hunts where students found objects matching vocabulary items, combining language practice with physical activity. Project-based learning requiring students to create posters, models, dramatizations engaged kinesthetic learners while developing multiple language skills simultaneously.

Content differentiation in EFL manifested through varied text types and difficulty levels. Teachers described maintaining topic consistency while varying text complexity. For example, when teaching environmental issues, teachers provided newspaper articles for advanced students, simplified informational texts for intermediate students, and heavily illustrated, vocabulary-supported materials for beginners. This approach ensured all students engaged with content while accessing it at appropriate linguistic and cognitive levels. Teachers emphasized importance of maintaining interest and relevance across difficulty levels rather than relegating struggling students to simplistic or juvenile materials.

Process differentiation involved varying instructional approaches and support structures. Teachers described providing graphic organizers for students needing structural support while allowing independent note-taking for others. The depth of direct instruction varied according to students’ needs, with extended explanations and multiple examples provided for those requiring intensive support and concise overviews offered to students who grasped concepts more quickly. Pacing flexibility allowed students to progress at appropriate rates, though teachers acknowledged difficulty managing this in whole-class contexts. Technology sometimes facilitated process differentiation through adaptive software providing individualized practice and immediate feedback.

Content and material diversity involved varied resources including authentic texts, digital platforms, visual aids, and hands-on materials. Teachers employed flexible grouping strategies, tiered assignments, and learning stations. Evaluation practices included projects, oral activities, portfolios, and self-assessment to make students’ diverse competencies visible. Classroom management strategies incorporated establishing routines, creating inclusive environments, and maintaining dynamic processes. Teachers positioned inclusion as fundamental, ensuring all students have equal learning opportunities in safe environments where they feel valued.

Portfolio assessment allowed students to demonstrate learning growth over time through collected work samples. Teachers described portfolios containing writing drafts showing revision processes, audio recordings documenting pronunciation improvement, and self-reflections on learning strategies. This longitudinal documentation helped students recognize progress even when current performance remained below grade-level expectations, maintaining motivation by highlighting individual growth rather than comparative standings.

Performance-based assessments included presentations, dramatizations, and project demonstrations allowing multiple skill integration. These authentic assessments valued creativity, cultural knowledge, and communication effectiveness beyond traditional test formats. One teacher described students creating travel brochures that required research, persuasive writing, visual design, and oral presentation, thereby assessing multiple competencies simultaneously while allowing differentiation in product format, topic choice, and level of complexity.

Grouping strategies varied based on learning objectives and student needs. Homogeneous grouping by proficiency level allowed teachers to provide targeted instruction matching students’ current abilities. For example, teachers created small groups for reading comprehension, assigning texts at appropriate difficulty levels while focusing instruction on specific comprehension strategies each group needed. Advanced students might analyze literary devices while beginners focused on main idea identification. This targeted approach enabled efficient use of instructional time while ensuring all students received appropriately challenging material.

Heterogeneous grouping promoted peer learning and scaffolding. Teachers paired stronger students with those needing support, explicitly teaching collaborative strategies to make peer assistance effective. In speaking activities, mixed-ability pairs allowed stronger speakers to model fluent expression while less proficient students gained confidence through supportive interactions. Teachers monitored these partnerships carefully, ensuring that assistance remained collaborative rather than one student simply providing answers.

Flexible grouping meant compositions changed based on tasks and purposes. For vocabulary activities, teachers might group students by learning style preferences (visual, auditory, kinesthetic). For project work, groups might form around student interests. For grammar practice, grouping by specific skill needs allowed focused intervention. This flexibility required careful planning and ongoing assessment to determine most appropriate grouping configurations for different instructional purposes.

### Effects on language development

Teachers reported three positive effect categories: learning process contributions, affective and motivational contributions, and academic achievement support. They observed DI enables students to learn at individual paces and capacities, making language learning more effective and learner-oriented. Multisensory activities increased learning permanence. Affectively, teachers reported that DI increased student interest, curiosity, engagement, and self-confidence. Teachers noted students became more willing to participate, reducing anxiety and creating comfortable learning atmospheres. Academic achievement appeared to improve, according to teachers’ observations, as students received appropriate challenge levels, with teachers observing enhanced performance across language skills.

Participants framed these effects in experiential rather than merely descriptive terms, reflecting the phenomenological depth of their accounts. One teacher articulated the motivational shift in the following way: “Before I started differentiating, some of my students had simply given up on English. They sat at the back and waited for the lesson to end. After I introduced tiered tasks, those same students started attempting work they would have refused before. Not because I lowered the bar, but because I placed the bar where they could actually see it” (T14). Another participant described a longer-term transformation in her class: “By the end of the semester, the gap between my strongest and weakest students had narrowed. Not because the stronger ones slowed down, but because the weaker ones had finally been given tasks they could engage with meaningfully” (T6). These accounts illuminate how DI reshaped the experiential landscape of language learning for both teachers and students.

The multisensory emphasis in implementation reflects strong grounding in learning style theory, though recent research questions rigid learning style categorizations (Pashler et al., 2008). While individuals may prefer certain modalities, exclusive reliance on preferred styles may limit learning. More effective approaches combine multiple modalities, allowing students to process information through varied channels regardless of preferences. Teachers’ integration of visual, auditory, and kinesthetic elements aligns with this multimodal perspective, providing varied access points benefiting all learners rather than attempting to match individual style preferences exclusively.

### Implementation challenges

Four challenge themes emerged: resource and time limitations, classroom structure and student factors, curriculum and system factors, and teacher competencies. Teachers cited large class sizes (typically 30–40 students) as major barriers making individualization difficult. Insufficient time for both planning and implementation limited differentiation possibilities. Limited materials, technology access, and physical space constraints restricted options. Curriculum rigidity, standardized testing pressures, and administrative demands constrained flexibility. Some teachers acknowledged insufficient DI knowledge and training, expressing needs for professional development in practical implementation strategies.

Teachers described feeling caught between recognition of DI benefits and practical realities limiting implementation. Large classes meant individualized attention remained minimal despite best intentions. One teacher articulated this tension with particular clarity: “I know what good differentiated teaching looks like. I have planned it, I have tried it. But when there are thirty-seven students in the room and the curriculum has set a pace I cannot deviate from, differentiation becomes something I do for 5 min at the end of the lesson if I am lucky” (T2). Time constraints meant most planning focused on core lessons rather than differentiated extensions. Teachers described feeling guilt about students whose needs went unmet despite theoretical commitment to individualized instruction.

### Teacher educators’ perspectives

Analysis of teacher educator interviews revealed five major themes: (a) conceptualization of DI, (b) methods used in teacher education, (c) pre-service teacher competencies and difficulties, (d) quality of preparation programs, and (e) implementation challenges.

#### Conceptualization of differentiated instruction

Teacher educators defined DI as an individualized, student-centered approach addressing cognitive, affective, and behavioral diversity. They emphasized three categories: meeting individual needs, supporting holistic development, and inclusive classroom culture. Educators stressed DI considers readiness, learning pace, interests, and learning styles systematically. They positioned DI as supporting not only academic achievement but also emotional well-being, motivation, and sense of belonging. Inclusive classroom culture was viewed as essential, requiring teachers to value diversity and create environments where all students feel respected and capable of success.

Teacher educators communicated these conceptualizations with a reflective orientation that itself reflected phenomenological intentionality. One participant described DI as inseparable from a particular educational worldview: “I tell my students that differentiated instruction is not a technique you add to your lesson plan. It is a commitment you make before you ever enter the classroom, a decision that every student in that room deserves to be seen” (A4). Another educator framed the concept in terms of professional identity formation: “Pre-service teachers who genuinely understand DI stop thinking of themselves as subject teachers and start thinking of themselves as learning architects. That shift is fundamental” (A2). These formulations suggest that for teacher educators, the meaning of DI was constituted not merely as pedagogical knowledge but as a professional-ethical orientation.

#### Methods in teacher education

Teacher educators employed three strategy categories: theoretical frameworks and modeling, active and experiential learning methods, and product-based applied studies. They integrated DI principles throughout curricula, modeling differentiation in their own teaching. Active learning methods included role-playing, simulations, case studies, and collaborative projects enabling pre-service teachers to experience DI from both teacher and learner perspectives. Product-based studies involved designing lesson plans, developing materials, and reflecting on applications. Educators emphasized practice-oriented and experience-based courses (teaching methods, language skills, teaching young learners) as most conducive to DI implementation, while theory-heavy courses (linguistics, material design) posed greater challenges due to rigid academic frameworks.

#### Pre-service teacher competencies and difficulties

Teacher educators assessed pre-service teachers’ DI competencies as developing but not yet mature. Student teachers demonstrated theoretical understanding struggled to translate knowledge into effective classroom practices into effective classroom practices. Difficulties included limited experience with diverse learner populations, insufficient practical application opportunities, and challenges balancing curricular demands with individualization. Educators emphasized needs assessment, flexibility, creativity, and reflective practice as essential competencies requiring stronger development through sustained practical experiences.

One educator described this theory-practice gap with precision: “They can write a lesson plan that includes differentiated tasks beautifully. But when I observe them in the classroom and a student responds unexpectedly, they freeze. They do not yet know how to differentiate in the moment, and that adaptive capacity is what real classrooms demand” (A6). Another participant noted that pre-service teachers often understood DI abstractly but had not experienced it themselves as learners, which limited their intuitive grasp of its purpose: “They have spent their entire education in rows, listening to a teacher explain things to the whole class. When I ask them to design for thirty different learners, they are working against 12 years of deeply embedded experience of what school looks like” (A3).

#### Quality of preparation programs

Teacher educators identified critical needs in program quality: curriculum integration and implementation-oriented education approaches. They emphasized DI should be structurally embedded in methodology and practicum courses rather than treated as supplementary topics. Practical lessons, scenarios, role-playing, reflective dialogue, and journals were recommended to support theory-practice integration. Educators stressed authentic learning experiences reflecting actual classroom diversity through scenario-based tasks, microteaching, and school-based practice opportunities to develop real teaching competencies beyond theoretical knowledge.

Teacher educators also discussed assessment challenges in evaluating pre-service teachers’ DI competencies. Traditional exams inadequately capture differentiation skills requiring practical application and adaptive decision-making. Performance-based assessments, such as lesson plan evaluations, teaching demonstrations, and reflective portfolios, provide stronger evidence of student learning but require substantial time for both development and evaluation. Several educators described using rubrics specifying DI components but acknowledged difficulty distinguishing between theoretical knowledge demonstration and actual implementation capacity. This assessment challenge reflects broader tensions in teacher education between measuring what can be easily assessed versus what matters most for effective teaching.

#### Implementation challenges and solutions

Teacher educators identified three challenge categories: program structure limitations, systemic and institutional obstacles, and faculty preparedness. Program limitations included insufficient practical application opportunities, time constraints, and limited resources. Systemic obstacles involved poor coordination between Council of Higher Education and Ministry of National Education, lack of clear policies supporting DI, and institutional disconnection between faculties and schools. Faculty preparedness challenges included educators’ own insufficient DI training and limited modeling of differentiated practices.

Solutions emphasized three areas: implementation strategies, programmatic improvements, and faculty development. Implementation solutions included needs analysis, practical examples, microteaching opportunities, and reflective practices. Programmatic solutions involved curriculum updates, professional development courses, strengthened university-ministry collaboration, and increased applied course integration. Faculty development solutions emphasized collaborative professional development, research-based practices, and ongoing training in contemporary DI approaches. Educators stressed effective DI implementation requires holistic systemic coordination beyond individual efforts.

## Discussion

The present study examined how secondary school EFL teachers and teacher educators in Türkiye understood and experienced differentiated instruction (DI), with particular attention to the relationship between teacher beliefs, perceived professional agency, teacher preparation, and institutional conditions. The findings revealed broad agreement between the two participant groups regarding the inclusive and learner-responsive purpose of DI. However, they also demonstrated a persistent gap between conceptual endorsement and practical enactment. This gap suggests that DI should not be understood solely as a collection of instructional techniques. Rather, it is a contextually negotiated pedagogical practice shaped by the interaction of teachers’ beliefs, adaptive decision-making, professional preparation, and the structural conditions of schooling.

### Differentiated instruction as a contextually negotiated practice

Both teachers and teacher educators conceptualized DI as a student-centered and inclusive approach designed to respond to differences in readiness, interests, language proficiency, learning pace, and support needs. This understanding is broadly consistent with Tomlinson’s (2001) framework, in which content, process, product, and learning environment are adapted in response to learner diversity. It also corresponds with research showing that teachers’ use of differentiation is influenced by their beliefs about learner differences and the instructional environments in which they work (Bi et al., 2023; Lindner et al., 2021).

The present findings extend this literature by showing that participants’ understandings of DI were not formed solely through formal pedagogical knowledge. Their accounts were shaped by repeated encounters with heterogeneous classrooms, disengaged learners, wide differences in language proficiency, and institutional expectations for uniform curricular coverage. DI therefore emerged as an evolving professional understanding rather than a fixed body of procedural knowledge. Participants did not merely describe differentiation as something they did; they described it as a way of interpreting learner difficulty and reconsidering professional responsibility.

This interpretation is particularly relevant from an educational psychology perspective. Teachers’ accounts suggested that the meaning they attributed to learner differences influenced how they interpreted student failure, motivation, participation, and instructional responsibility. Rather than locating difficulty exclusively within the learner, several participants described reconsidering the design of instruction itself. Such accounts position teacher beliefs as an important psychological mechanism through which instructional adaptation becomes possible.

Participants also framed DI as an ethical commitment to inclusion and educational equity. This framing moves beyond academic access by incorporating students’ sense of belonging, confidence, participation, and emotional safety. It is consistent with contemporary inclusive pedagogy, which emphasizes that inclusive teaching practices are related not only to academic participation but also to students’ social and emotional inclusion (Alnahdi et al., 2022). In this sense, DI was experienced as both a pedagogical and professional-ethical orientation.

### Teacher beliefs, professional agency, and structural constraints

Although participants expressed strong support for DI, their ability to implement it consistently was constrained by large classes, limited planning time, curriculum rigidity, insufficient resources, and examination pressures. These findings align with previous research identifying class size, time limitations, standardized curricula, and restricted institutional support as major barriers to differentiated practice (Goddard et al., 2015; Suprayogi et al., 2017; Bondie et al., 2019; Grecu, 2023).

The contribution of the present study lies in demonstrating how these barriers shape teachers’ perceived professional agency. Participants generally did not describe unwillingness to differentiate. Instead, they described situations in which they understood what responsive instruction required but lacked sufficient time, flexibility, or organizational support to act on that understanding. The central difficulty was therefore not simply a lack of pedagogical knowledge but a mismatch between professional intention and perceived capacity for action.

This finding supports the view that DI implementation develops through the interaction of teacher beliefs, perceived competence, motivation, classroom heterogeneity, and institutional support (Bi et al., 2023; Kalinowski et al., 2024). Teachers may value differentiation and possess relevant knowledge while still feeling unable to enact it consistently. Professional agency in this context should therefore not be treated as an individual disposition isolated from working conditions. It is enabled or constrained by the organizational and policy environments in which instructional decisions are made.

Participants associated DI with increased student motivation, participation, confidence, and engagement. These outcomes were reported as teacher perceptions rather than directly measured student effects; nevertheless, they suggest that differentiation may influence students’ psychological relationship with language learning. Providing tasks at accessible yet challenging levels may support perceptions of competence, while meaningful choices and flexible participation structures may support autonomy and engagement. These observations are compatible with motivational perspectives emphasizing competence, autonomy, and relatedness as foundations of engagement (Deci and Ryan, 2000).

The findings should not, however, be interpreted as evidence that DI automatically improves motivation or achievement. The study examined participant experiences and beliefs rather than measured psychological or academic outcomes. Its contribution is to show how teachers themselves understood the affective consequences of pedagogical responsiveness and how these perceived consequences strengthened their commitment to differentiation.

### Teacher preparation and the theory–practice gap

Teacher educators reported that pre-service teachers often understood DI conceptually but struggled to make adaptive decisions in authentic classroom situations. This finding reflects a distinction between declarative knowledge of differentiation and the capacity to modify instruction responsively when students react in unexpected ways. The latter requires adaptive professional judgment developed through repeated opportunities to design, enact, observe, and reflect on instruction.

Previous research has similarly shown that effective preparation for DI requires more than isolated theoretical coursework. Teacher educators need to model differentiated practice, connect it with inclusive frameworks, and provide sustained opportunities for enactment and reflection (Coubergs et al., 2017; Griful-Freixenet et al., 2021; Nketsia et al., 2024). Longitudinal, collaborative, and practice-oriented professional learning is also more likely to support implementation than short-term knowledge transmission (Langelaan et al., 2024).

The present study adds a contextual dimension to this argument. International DI frameworks often assume a degree of curriculum flexibility, planning time, resource availability, and small-group organization that may not be readily available in Turkish secondary schools. Pre-service teachers may therefore be prepared for idealized differentiation rather than for differentiation under actual constraints. Teacher preparation should consequently include scenarios that require prospective teachers to make adaptive decisions in large, mixed-proficiency, examination-oriented classrooms.

This interpretation reframes teacher readiness as context-specific adaptive capacity rather than the simple acquisition of universal instructional competencies. Readiness for DI involves understanding its principles, but it also requires the ability to negotiate curriculum demands, student diversity, limited resources, and institutional expectations. Practice-based experiences such as microteaching, extended practicum placements, lesson-study activities, case analysis, and reflective portfolios may provide opportunities to develop this capacity.

Teacher educators’ own preparedness also requires attention. Participants acknowledged variation in the extent to which faculty members modeled differentiated practices. This finding suggests that reform cannot be directed only toward pre-service teachers. Teacher educators require professional learning opportunities that enable them to model DI, design authentic learning experiences, and assess adaptive teaching competence through performance-based methods rather than conventional examinations alone.

### Educational psychology implications

The findings contribute to educational psychology by illustrating how teacher beliefs, motivation, perceived agency, and institutional conditions jointly shape adaptive instruction. DI implementation was not determined by knowledge alone. It emerged from teachers’ interpretations of learner diversity, their confidence in making instructional adaptations, their motivation to respond to individual needs, and their perceptions of whether the school environment permitted such decisions.

This perspective supports a move away from deficit explanations that attribute weak implementation primarily to teacher resistance or insufficient competence. Even well-prepared teachers may struggle to differentiate when curriculum pacing, class size, assessment systems, and resource conditions restrict professional judgment. Conversely, supportive organizational conditions may strengthen teachers’ willingness to experiment, reflect, and persist with adaptive practices.

The study therefore conceptualizes DI as a psychologically and institutionally situated form of professional action. Teacher beliefs provide the interpretive basis for recognizing learner diversity; professional agency supports adaptive decision-making; motivation sustains the additional effort required for planning and monitoring; and institutional conditions determine the extent to which these psychological resources can be translated into classroom practice. This integrated interpretation extends traditional DI frameworks by positioning implementation within the reciprocal relationship between individual and contextual factors.

### Implications for teacher education and practice

The findings suggest that DI should be integrated across teacher education programs rather than presented as an isolated topic. Methodology and practicum courses should provide repeated opportunities for pre-service teachers to analyze learner variability, design tiered and flexible tasks, implement differentiated lessons, and reflect on how their decisions affect motivation, participation, and access to learning. Teacher educators should model DI within university courses so that prospective teachers experience responsive instruction as learners as well as study it conceptually. This recommendation is consistent with research emphasizing modeling, enactment, and structured reflection in DI preparation (Coubergs et al., 2017; Griful-Freixenet et al., 2021; Nketsia et al., 2024).

In-service professional development should be sustained, collaborative, and closely connected to classroom practice. One-time workshops are unlikely to develop the adaptive decision-making required for heterogeneous EFL classrooms. Coaching, peer observation, collaborative lesson planning, and professional learning communities may support teachers in translating principles into context-sensitive practices. This approach is consistent with evidence that effective DI professional development is longitudinal, active, and collaborative (Langelaan et al., 2024).

School–university collaboration should also be strengthened. Joint practicum design, extended school placements, mentoring partnerships, and collaborative inquiry projects could reduce the discontinuity between teacher preparation and classroom realities. Coordination between the Council of Higher Education and the Ministry of National Education may help align teacher education, curriculum expectations, and professional development priorities.

At the school and policy levels, expectations for DI should be accompanied by enabling conditions. Teachers require adequate planning time, access to varied instructional materials, manageable classroom structures, and sufficient curricular flexibility. Policies that promote differentiation without addressing these conditions risk positioning implementation as an individual teacher responsibility. The findings instead suggest that responsive teaching should be regarded as a shared organizational responsibility involving teachers, teacher educators, school leaders, and policymakers.

Technology may support DI through adaptive tasks, varied learning resources, and flexible forms of student participation. However, the findings do not support technology use as an end in itself. Professional learning should focus on how digital tools can be aligned with specific differentiation goals. In this sense, technological pedagogical content knowledge should be developed as an integrated capacity for selecting tools according to learner needs and instructional purposes (Koehler and Mishra, 2009).

### Limitations and future research

Several limitations should be considered when interpreting the findings. First, the study included teachers and teacher educators from two provinces in Türkiye. Purposeful sampling enabled detailed examination of participants with relevant experience, but the findings should not be treated as representative of all Turkish EFL contexts. Regional, socioeconomic, urban–rural, and school-type differences may shape opportunities for DI in different ways.

Second, the study relied primarily on self-reported experiences. Interviews provided access to participants’ beliefs, interpretations, and perceived constraints, but they did not establish whether reported practices were consistently enacted in classrooms. The positive effects attributed to DI, including increased motivation, participation, confidence, and achievement, represent participant perceptions rather than directly measured outcomes.

Third, the cross-sectional design captured experiences at one point in time. It did not examine how teacher beliefs, professional agency, or DI practices change through experience, professional learning, or institutional reform. Student perspectives were also not included, limiting the study’s ability to compare teacher interpretations with learners’ experiences.

Future research should combine interviews with classroom observations, lesson artifacts, student perspectives, and measures of engagement or achievement. Longitudinal studies could examine how pre-service teachers’ adaptive capacity develops across coursework, practicum, and early career teaching. Intervention research could test practice-based professional development models involving coaching, collaborative planning, and reflective inquiry. Comparative studies across regions, school types, and educational levels would help clarify how institutional context influences the relationship between teacher beliefs and DI implementation. Research should also examine how technology-supported differentiation affects student engagement and learning under conditions of unequal digital access.

## Conclusion

This study shows that EFL teachers and teacher educators in Türkiye broadly understand differentiated instruction as an inclusive and learner-responsive approach. However, conceptual understanding does not automatically translate into consistent implementation. Participants described a persistent mismatch between their pedagogical intentions and the institutional conditions within which they worked.

The findings advance current understanding by conceptualizing DI as a contextually negotiated practice shaped by the interaction of teacher beliefs, perceived professional agency, motivational commitment, teacher preparation, and systemic conditions. This interpretation shifts attention away from explanations centered solely on individual teacher competence and toward the psychological and organizational conditions that enable adaptive teaching.

For teacher education, the central implication is that readiness for DI should be developed as context-specific adaptive capacity. Prospective and practicing teachers need opportunities to make, test, and reflect on instructional decisions in conditions resembling authentic EFL classrooms. Sustainable implementation also requires institutional environments that protect professional judgment, provide practical support, and treat responsiveness to learner diversity as a shared responsibility rather than an individual obligation.

## Data Availability

The data are available from the corresponding author upon request.
